# Automated White Matter Hyperintensity Segmentation Using Bayesian Model Selection: Assessment and Correlations with Cognitive Change

**DOI:** 10.1007/s12021-019-09439-6

**Published:** 2020-02-15

**Authors:** Cassidy M. Fiford, Carole H. Sudre, Hugh Pemberton, Phoebe Walsh, Emily Manning, Ian B. Malone, Jennifer Nicholas, Willem H Bouvy, Owen T. Carmichael, Geert Jan Biessels, M. Jorge Cardoso, Josephine Barnes, for the Alzheimer’s Disease Neuroimaging Initiative

**Affiliations:** 1 Dementia Research Centre, Department of Neurodegenerative Disease, UCL Queen Square Institute of Neurology, London, UK; 2 School of Biomedical Engineering and Imaging Sciences, King’s College London, London, UK; 3 Department of Medical Physics and Biomedical Engineering, University College London, London, UK; 4 London School of Hygiene and Tropical Medicine, London, UK; 5 Department of Neurology and Neurosurgery, Brain Center Rudolf Magnus, University Medical Center Utrecht, Utrecht, the Netherlands; 6 Pennington Biomedical Research Center, Baton Rouge, LA USA

**Keywords:** White matter hyperintensities, Automated segmentation, Magnetic resonance imaging, Neurodegeneration, Vascular pathology, Alzheimer’s disease

## Abstract

Accurate, automated white matter hyperintensity (WMH) segmentations are needed for large-scale studies to understand contributions of WMH to neurological diseases. We evaluated Bayesian Model Selection (BaMoS), a hierarchical fully-unsupervised model selection framework for WMH segmentation. We compared BaMoS segmentations to semi-automated segmentations, and assessed whether they predicted longitudinal cognitive change in control, early Mild Cognitive Impairment (EMCI), late Mild Cognitive Impairment (LMCI), subjective/significant memory concern (SMC) and Alzheimer’s (AD) participants. Data were downloaded from the Alzheimer’s disease Neuroimaging Initiative (ADNI). Magnetic resonance images from 30 control and 30 AD participants were selected to incorporate multiple scanners, and were semi-automatically segmented by 4 raters and BaMoS. Segmentations were assessed using volume correlation, Dice score, and other spatial metrics. Linear mixed-effect models were fitted to 180 control, 107 SMC, 320 EMCI, 171 LMCI and 151 AD participants separately in each group, with the outcomes being cognitive change (e.g. mini-mental state examination; MMSE), and BaMoS WMH, age, sex, race and education used as predictors. There was a high level of agreement between BaMoS’ WMH segmentation volumes and a consensus of rater segmentations, with a median Dice score of 0.74 and correlation coefficient of 0.96. BaMoS WMH predicted cognitive change in: control, EMCI, and SMC groups using MMSE; LMCI using clinical dementia rating scale; and EMCI using Alzheimer’s disease assessment scale-cognitive subscale (*p* < 0.05, all tests). BaMoS compares well to semi-automated segmentation, is robust to different WMH loads and scanners, and can generate volumes which predict decline. BaMoS can be applicable to further large-scale studies.

**Electronic supplementary material:**

The online version of this article (10.1007/s12021-019-09439-6) contains supplementary material, which is available to authorized users.

## Introduction

Cerebral small vessel disease is an important cause of cognitive decline. White matter hyperintensities of presumed vascular origin (WMHs) can be detected on magnetic resonance imaging (MRI) using T2-weighted Fluid Attenuated Inversion Recovery (FLAIR) sequences, and represent one type of cerebrovascular damage which are common in Alzheimer’s disease (AD). WMHs are associated with brain atrophy (Barnes et al. 2013; Fiford et al. 2017) and cognitive decline (Carmichael et al. 2010), but their mechanistic role in AD is unknown (Prins and Scheltens 2015). As AD is an insidious, multifactorial syndrome, which is highly variable from person to person, efforts are therefore turning towards large-scale clinical data sets to provide insights into disease mechanisms and progression (Masters et al. 2015). Examples of these datasets are the Alzheimer’s disease Neuroimaging Initiative (ADNI) and UK Biobank, which include demographic information, magnetic resonance images (MRI) and many more variables. Big data may revolutionise AD research and our understanding of WMHs, however the size of these datasets necessitates the use of automated methods to derive new variables from MRI, such as WMH volume. Drawing correct inferences relies on choosing a well validated method that is reliable, accurate, and adaptable to data from multiple sites. If we succeed, WMH from large-scale datasets may prove valuable in understanding whether WMHs play a causative role in cognitive impairment and dementia, and the mechanisms underlying this link. In this paper, we assess a WMH segmentation method, Bayesian Model Selection (BaMoS) (Sudre et al. 2015), against human rater estimates (rater guided semi-automated segmentation) of WMH and test whether BaMoS derived WMH measurements can predict change in neuropsychological test scores.

Classification of WMHs is complex, as, whilst they are clearly visible on MRI in many individuals, they are extremely heterogeneous in nature. They range from large confluent WMH deep in the white matter (WM), to spherical punctate lesions and periventricular lesions. Manual delineation of WMH is one method to assess WMH. However, this process is time-consuming, requires training and is still variable in volume estimation, especially in areas of diffuse WMH. Whilst manual delineation is impractical for large scale studies, such segmentations provide essential ‘gold standards’ against which to test automated WMH segmentation methods. Manual segmentations are important because humans are able to easily identify bright regions of artefact, which FLAIR imaging is especially susceptible to (Bakshi et al. 2000). However, human-generated WMH estimates remain liable to inconsistencies and error; therefore in order to ensure an algorithm is properly assessed, it is important to compare it to a reliable and meticulously generated manual standard, from multiple raters segmenting sufficient numbers of individuals. Techniques which incorporate human decision making and computerised thresholds to automatically draw boundaries are useful in speeding up the manual segmentation process. Such a technique is used in this study as a gold standard: the technique uses both intensity thresholds and manual decision making and delineation as part of the protocol. As this operator-intensive technique used is not fully manual it is referred to as ‘semi-automated’.

No automated WMH segmentation method is likely to be completely accurate, so it is essential to quantify the measurement error though comparison to a gold standard and assess whether the expected associations are detected between WMH volume and clinically relevant measures such as neuropsychological test scores. Numerous automated WMH segmentation methods exist, and each has their own strengths and limitations (Caligiuri et al. 2015; Dadar et al. 2017). In this study, we assess BaMoS, which has previously compared well to a small number of gold standard human estimates, and to a larger dataset of existing automated segmentations (Sudre et al. 2015). BaMoS has undergone methodological improvements since 2015, and this paper serves as a better, more in-depth assessment of the algorithm. We include a larger set of gold standards produced by multiple raters, an extensive examination of errors, and an assessment of the cognitive associations of BaMoS-generated WMH volumes. First, we will explore whether BaMoS performs well against human semi-automated estimates of WMH volume in a subset of 60 ADNIGo and ADNI2 control and AD patients. Secondly, we will investigate whether BaMoS-generated WMH volumes are associated with changes in cognition in a large set of ADNIGo and ADNI2 controls, early MCI (EMCI), late MCI (LMCI), Subjective/Significant Memory Concern (SMC) and AD patients. We chose to use newly-enrolled subjects from ADNIGo (EMCI) and ADNI2 (controls, SMC, EMCI, LMCI and AD) datasets since the same imaging protocols are used for these two phases of ADNI and together these datasets encompass individuals from normal aging through to clinical AD. The purpose of this work is threefold: i) present a robust semi-automated protocol for the segmentation of WMH; ii) evaluate an existing automated WMH segmentation algorithm against this new gold standard; iii) validate the possibility of applying the automated WMH segmentation algorithms to large-scale studies.

## Methods

### Participants

Data used in this study were obtained from the Alzheimer’s Disease Neuroimaging Initiative (ADNI) database (http://www.loni.usc.edu/). Data from phases ADNIGo and ADNI2 were used in this paper. Launched in 2003, ADNI is a multicentre, private/public funded longitudinal study investigating healthy adults, MCI and AD patients, and is led by Principle Investigator Michael W. Weiner, MD. Its primary goal is to test whether serial magnetic resonance imaging (MRI), positron emission tomography (PET), other biological markers, and clinical and neuropsychological assessment can be combined to measure AD progression. For up-to-date information, see www.adni-info.org.

Written informed consent was obtained as approved by the Institutional Review Board at each participating centre. Participants took part in baseline clinical, neuropsychometric and MRI assessments, and periodical assessments thereafter, the frequency of which varied dependent on the diagnostic group. To assess how BaMoS estimated WMH affects longitudinal cognitive change the Mini-Mental State Examination (MMSE), Clinical Dementia Rating (CDRGlobal), Trails A (a measure of processing speed), Trails B (a measure of executive functioning) and the Alzheimer’s disease Assessment Scale cognitive subscale (ADAS-Cog) were investigated (please see http://adni.loni.usc.edu/wp-content/uploads/2008/07/ADNI_GO_Procedures_Manual_06102011.pdf and https://adni.loni.usc.edu/wp-content/uploads/2008/07/adni2-procedures-manual.pdf).

For semi-automated protocol development, MRI from 20 type 2 Diabetes and control patients were supplied by Utrecht University. WMH had been segmented on these scans already and therefore provided a dataset against which the semi-automated protocol could be assessed. All patients were over the age of 50 and had varying burdens of WMH (Reijmer et al. 2013). This study was approved by the medical ethics committee of the University Medical Center Utrecht, the Netherlands, and written informed consent was obtained from all participants.

### Image Acquisition and Assessment

All baseline 3T T1-weighted and 3T T2-weighted FLAIR images were downloaded for ADNIGo and ADNI2 patients on 6th November 2014. The ADNI MRI protocol is described in detail elsewhere (Jack et al. 2008). Axial 3T FLAIR was acquired with voxel sizes of 0.85994 × 0.8594 × 5 mm. Following acquisition, each image underwent quality control at the Mayo Clinic (Rochester, MN) which included protocol compliance check, inspection for clinically significant medical abnormalities, and image quality assessment.

For semi-automated segmentation, T1-weighted images were registered to T2 FLAIR images, as WMH are clearer, and more easily viewed on T2 FLAIR. T1-weighted images were co-registered to FLAIR using Reg-Aladdin in NiftyReg (https://github.com/KCL-BMEIS/niftyreg) (Modat et al. 2014). All FLAIR images for semi-automated segmentation, including the protocol adaptation, training and test sets were visually assessed for motion and significant artefact. NiftyMIDAS software was used for segmentation, allowing simultaneous viewing and segmentation of the FLAIR and T1-weighted image (T1 co-registered to FLAIR). NiftyMIDAS has recently been made open source as part of NifTK (https://github.com/NifTK/NifTK) (Clarkson et al. 2015).

Utrecht images were acquired on a 3 T Philips scanner, voxel sizes for FLAIR were 0.958 × 0.958 × 3 mm. The 3D T1-weighted scan was registered to the T2 FLAIR. All images were bias corrected. Semi-automated WMH segmentations were produced by trained raters.

## Semi-Automated Segmentation

### Initial Protocol Development (Utrecht Scans)

For this study, a semi-automated WMH segmentation protocol was developed to provide a human-derived gold standard for WMH segmentation. The segmentation process is referred to as ‘semi-automated’ (rather than manual) due to the use of computerised thresholds for segmentation, whereby a given voxel is included if it exceeds a predetermined intensity value (% of median brain intensity). Raters must decide which voxels are considered as WMH, by placing a threshold ‘seed’ in a voxel of a lesion considered to be WMH by the rater. The extent of the lesion is then determined by the thresholds and manual interventions by removing voxels which are erroneously segmented as WMH by the thresholds.

Initial rules for thresholds and classification of WMH were developed by referring to an existing set of manual WMH segmentations from type 2 Diabetes patients and controls scanned in Utrecht. These 20 segmentations were viewed on their corresponding FLAIR and T1-weighted images. Inspection of these manual WMH segmentations and consultation of the literature, led to rules for the location and appropriate thresholds for WMH delineation, as well as window values for viewing scans. General rules included ensuring potential voxels of WMH were hyperintense on FLAIR and hypointense on T1, dismissing artefact (by consulting both T1 and T2 FLAIR), and avoiding commonly hyperintense areas (including the corticospinal tracts, normal appearing septal and corpus callosal regions and normal appearing corona radiata, in addition to posterior regions of the frontal horn of the lateral ventricles) (Gawne-Cain et al. 1997; Wardlaw et al. 2015).

### Segmentation of ADNI2/go

**Fig. 1:**
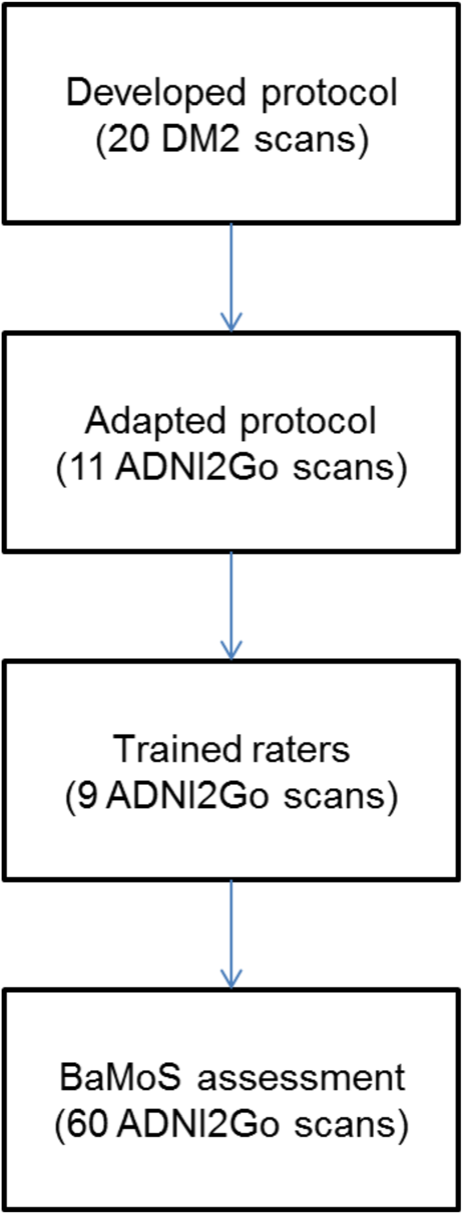
Flowchart of process from initial protocol (developed on 20 Diabetes Mellitus and controls subjects from Utrecht (DM2)), through to BaMoS segmentation assessment set. At each stage different subjects’ scans were used. ADNI2/Go = Alzheimer’s Disease Neuroimaging Initiative Phases 2 and GO.

A total of 80 scans were used in the ADNI2/Go semi-automated segmentation stage. There were three phases to the ADNI2/Go semi-automated segmentation process; adaptation of the protocol to ADNI2/Go scans (11 unique scans), training raters (9 unique scans), and segmentation of the assessment set by all raters (60 unique scans), see Fig. 1. There were 4 raters. At each stage of the semi-automated segmentation process (protocol adaptation, training raters and automated assessment) different scans were used in each set of images.

### Adaptation of Protocol

The protocol required adaptation for ADNIGo and ADNI2 images, due to the thicker T2 FLAIR slices, and some ventricular ghosting artefacts. ADNI scans were also acquired at multiple sites using different scanners, as opposed to the Utrecht dataset. Multiple scanner models exist across the sites in ADNI. For the methods comparison we chose to restrict the sample segmented to the most popular model types from the three main ADNI scanner manufacturers, Signa HDxt (General Electric (GE) medical systems), “Achieva” (Philips) and “Triotim” (Siemens). To adapt the protocol from Utrecht scans to ADNI2/Go, 11 scans (5 GE, 3 Philips and 3 Siemens) were used. These 11 participants were chosen to reflect a variety of WMH loads using WMH values from University California Davis (http://www.loni.usc.edu/ see ADNI2_Methods_UCD_WMH_Volumes_20131218.pdf).

Following ADNI pre-processing of the T1, and registration of T1s to FLAIR space (both detailed above), the images for semi-automated segmentation were not processed any further. Raters began segmentation without prior information, other than thresholds required for segmentation. The defining characteristics of this semi-automated protocol were the use of two thresholds (defined as a % of median brain intensity), with a higher intensity threshold for WMH with uncertain fuzzy boundaries, and a lower threshold for WMH with definite boundaries. The higher threshold was used in areas where WMH are likely to be developing, for WMH which may have a bright core, surrounded by diffuse (possibly developing) hyperintensity, and so-called confluent hyperintensities. High thresholds were also used in regions where some hyperintensity is considered clinically normal (e.g. periventricular caps and corticospinal tracts) (Gawne-Cain et al. 1997). A lower threshold was used for lesions that are less bright, which have defined boundaries, so-called punctate WMH. A manual freehand approach with no threshold was also possible in cases where the rater was certain of a WMH, but which was not picked up by the thresholds. In such cases where abnormal signal was apparent on both imaging modalities (T1 and FLAIR), extra care was taken to avoid temporal lobe artefacts and vascular flow artefacts. Thresholds were based on the median whole brain intensity, which was calculated using a brain mask generated using BMAPS on the T1-weighted image and then copied to the FLAIR image (Leung et al. 2011). BMAPS is a multi-atlas automated brain segmentation tool which identifies brain images from a template library which match well to the novel target. The templates are propagated, thresholded and fused together to create an automated brain segmentation on the target image.

A high (low) threshold was set at 130% (120%) of median brain intensity for Siemens and Philips. GE scanners tended to produce scans with a hyperintense posterior brain region; therefore for this scanner type the thresholds were increased to 145% and 130%. For viewing FLAIR scans maximum viewing intensities were 238% for Siemens and Philips and 340% for GE scanners.

### Choice of Segmentation Assessment Set

The assessment set was composed of 60 individuals, 30 controls and 30 AD patients, who had not been used during protocol development. There were 10 controls and 10 AD patients from each of the three scanner types (Siemens, Philips and GE scanner).

The assessment set was segmented by 4 raters after being trained by practicing on 9 images. Rater 1, who developed the protocol, trained raters 2 and 3. Rater 2 trained rater 4. Raters were blinded to each other’s segmentations. Raters were accepted when they segmented with a mean volume difference of less than 15% compared to the reference (rater 1) on the training set of 9 images, inclusive of small volumes. All raters were accepted. The assessment set was blinded to raters in terms of the subject identity, diagnosis, other raters’ segmentations and scanner type. Rater 1 segmented the dataset again to obtain intra-rater reliability.

### Consensus Segmentations

Consensus segmentations were generated using combinations of rater segmentations. These consensus segmentations were used to look for specific differences in each rater’s performance, for example, rater 1 would be compared to a consensus combination of 2, 3, and 4; rater 2 would be compared to a consensus of 1, 3 and 4, etc. A consensus combination of all 4 raters’ segmentations was used for the comparison with BaMoS. Consensuses were generated using majority voting: first, majority voting was used for consensus sets of three out of four raters (raters 1–2-3, raters 1–2-4, raters 2–3-4 and raters 1–3-4), then majority voting using each of the 4 consensus sets were obtained for the overall consensus.

### Automated Segmentation

Supratentorial WMH were estimated on the baseline scan. WMH were segmented jointly using T1-weighted and T2-FLAIR sequences rigidly coregistered in T1 space. BaMoS models the data as a multivariate mixture of Gaussians and can be used for segmentation of pathological tissue types (e.g. WMH). The model is able to jointly model normal and unexpected observations. Each anatomical tissue class (grey matter, white matter, cerebrospinal fluid, non-brain) is modelled as a mixture of Gaussians whose number is automatically and dynamically determined using a split and merge strategy. Both skull-stripping and atlases are obtained as a result of the label-fusion GIF framework (Cardoso et al. 2015). Skull stripping was incorporated in the automated segmentation as part of the BaMoS pipeline. Furthermore, a bias field correction is applied at the initialisation step of the split and merge process following the additive modelling described by Van Leemput et al (Van Leemput et al. 1999). In order to enhance sensitivity to the outliers, an initial outlier map is derived after convergence of the initial (1 component per tissue class) Gaussian mixture model. This enhancement of sensitivity was added to the initial BaMoS method and presented in its longitudinal extension (Sudre et al. 2017). After convergence of the model, candidate lesion voxels are selected from the outlier part of the model based on their “outlierness” (distance from normalcy) compared to healthy (inlier) white matter. Correction for false positives was lastly automatically applied to discard regions of muscle, fat, skin, choroid plexus or other wrongly classified tissue based on clinical knowledge of WMH. In order to increase sensitivity to smaller WMH lesions where partial volume effect was more likely to happen, a two-step, two-threshold detection of candidate lesions was adopted: after the use of the initial threshold for lesion outlierness to detect the bulk of the lesions, a lower threshold (2/3 of initial value) was further used to include small clusters of hyperintensity (less than 60 voxels). Furthermore, the classification of candidate lesion connected components was performed in two consecutive steps: first with a 18 neighbourhood followed by a 6 neighbourhood definition in order to avoid discarding regions of mixed origin (artefacts + true lesion). Specific, user-defined BaMoS parameters, and software needed to produce segmentations reported, are listed in the supplementary section.

All WMH segmentations were checked by a trained WMH rater for gross errors. This quality control step was used to further develop robust improvement to the post-processing step, contributing to a better handling of artefacts and their differentiation from true WMH. Where there were image duplicates the best segmentation was chosen.

BaMoS segmentations are run in T1 space and volumes obtained by integrating the probabilistic map of WMH. The semi-automated segmentations are performed in FLAIR space. To account for this, we re-ran BaMoS segmentations in T2 FLAIR space to generate binarised segmentations for the 60 individuals in the assessment set that would be comparable to the semi-automated segmentations. The assumption that T1 space segmentations generated in the full dataset would be more-or-less equivalent to the T2 FLAIR segmentations from the assessment set was tested (see below, statistics section).

## Statistical Methods

### Group Demographics

Stata SE v13 (Stata Corp.) was used to perform statistical tests and analyse data. To look for differences in baseline variables between diagnostic groups, linear regression was used for continuous variables. Median WMH volumes were reported from T1 space BaMoS segmentations, as opposed to T2 FLAIR space segmentations (see above). To look for differences between groups in sex and presence of an APOE ε4 allele, Fisher’s exact test was used. For group differences in race, Pearson’s chi squared was used. Demographics were also assessed in the subset used for the WMH methods comparison.

### Assessment of Semi-Automated Segmentation and BaMoS

We performed two sets of analyses to examine the agreement between WMH volumes resulting from raters using semi-automated segmentation. Firstly, we assessed intra-rater reliability from the intraclass correlation coefficient (ICC) for agreement between the two sets of segmentations by rater 1. Then we assessed the inter-rater reliability by calculating the ICC comparing rater 2, 3 and 4’s segmentations with rater 1, and then by comparing each rater to consensus combinations of the remaining raters’ segmentations. To compare volumes between raters, WMH volumes were log transformed to the base 2 (log_2_WMH), as WMH values were skewed. A paired t-test was used to compare the mean log-transformed volume between raters compared to rater 1.

To assess the agreement between semi-automated segmentation and BaMoS, we calculated the intraclass correlation coefficient (ICC) for agreement between BaMoS and each rater’s semi-automated segmentation, and to a consensus combination of the four raters. To compare volumes between raters and BaMoS, WMH volumes were log transformed to the base 2 (log_2_WMH), as WMH values were skewed. A paired t-test was used to compare the mean log transformed volume from BaMoS with each rater’s. Similarly, a paired t-test was used to compare BaMoS and the consensus segmentation of all four raters. We used a Bland-Altman plot to graphically compare BaMoS’ WMH estimates with the estimates of rater 1; the difference between the volumes from the two techniques was plotted against the average of the two volumes. The mean difference between the two techniques, and the 95% limits of agreement (mean difference ± 1.96*standard deviation of differences), were also calculated and plotted.

In order to evaluate overlap between segmentations, we used the Dice score coefficient (DSC) expressed as the ratio between twice the volume of overlap and the sum of segmented volumes. To further understand the origin of disagreement between segmentations, discrepancies were separated into two categories following the description given by Wack et al. 2012 (Wack et al. 2012). Detection error (DE) corresponds to the volume of segmented lesion for which the full extent of the corresponding connected component of segmented voxels is completely absent from one of the compared segmentations. Outline error (OE) corresponds to differences in the specific voxels segmented within the same lesion between two segmentations. For a given cluster of connected lesion voxels, the lesion is considered the same across the two segmentations if their intersection is not empty: they share at least one segmented voxel. DE and OE were further divided into false positives (FP) and false negatives (FN) for completeness of the assessment. Details and illustration of these subtypes of error can be found in the first section of the online resource and online tools that can be used to compare segmentations are listed in the supplementary section.

We compared the volumes between BaMoS-generated WMH estimated in T1 space and BaMoS-generated WMH estimated in FLAIR space. We log-transformed WMH volumes to the base 2, and assessed whether there were differences in mean volumes using a t-test. The intra-class correlation coefficient was used to assess correlation between the two methods.

Lastly, we investigated differences in BaMoS’ performance according to different scanner types. Using linear regression, we modelled each overlap metric separately (Dice, OEFP, OEFN, DEFN, DEFP), predicted by scanner type (Philips, Siemens or GE) and adjusted for each rater providing the reference segmentation (rater 1, rater 2, rater 3, rater 4, consensus).

### Diagrammatic Representation of WMH Regional Distribution

In order to represent the regional distribution of WMH, the white matter and deep grey matter volume was divided into 36 regions using the method described in (Sudre et al. 2018). In a first stage, the volume encompassed between the ventricular surface and the cortical sheet is divided into equidistant layers using the solution to the Laplace equation solved on this volume. Second, the lobar parcellations of the gray matter obtained from the GIF software are propagated onto the white matter +deep grey matter volume to separate the region into lobes. Basal ganglia, thalamic and infratentorial regions are considered separately. The layer and lobar divisions lead to a total of 36 regions (4 × 9) that were then used to visually represent the spatial distribution of WMH differences between BaMoS and the consensus in the shape of bullseye plots. In these plots, each angular segment corresponds to a different lobar region while the concentric layers represent the equidistant extracted layers with the distance from the ventricular surface increasing with the radius. To represent the spatial distribution of differences between BaMoS and the consensus, OEFP, OEFN, DEFP and DEFN were displayed in the bullseye plots as proportions of total error and as proportions of true positive WMH volume. To further illustrate locations of errors, difference maps were overlaid on images randomly selected from individuals with a low (<2 ml), medium and high (>6 ml) WMH load.

### Associations of BaMoS Derived WMH to Baseline, and Change in Neuropsychology

We fitted multilevel linear mixed-effects regression models for repeated measures of cognition (MMSE, ADAS-Cog, Trails A, Trails B and CDRGlobal). We used the global score of the Clinical Dementia Rating, and the total 13 elements of the ADAS-Cog. Interval in years between baseline scan and each cognitive examination date was included as a fixed effect, in order for the resulting coefficient to represent change in cognition per year (outcome). Models were fitted separately in each diagnostic group. Covariates were included as main effects and as interaction terms with interval. These included WMH, age, sex, years of education, APOE ε4 carrier status (presence/absence of an ε4 allele) and race, similarly to Carmichael et al. (2010). The fitting of the models in this manner allowed these covariates to affect mean MMSE and how this changed over time. Race was a binary covariate coding white race vs non-white race; there were insufficient numbers to investigate the effect of each race on the outcome. Models were run using log_2_WMH as a predictor. Participant-level random effects for intercept and time since baseline MMSE measurement were included to permit between-participant heterogeneity in baseline MMSE and between-participant heterogeneity in change in MMSE. Models were run using other neuropsychological tests in place of the MMSE (ADAS-Cog, Trails A, Trails B and CDRGlobal). Unstructured covariance of the random effects was used to allow for a correlation between baseline psychological test score and rate of change in score. A separate residual variance was fitted for each diagnostic group.

## Results

### Participants

Of 1010 downloaded ADNI scans, 10 failed WMH segmentation quality control, 2 had incomplete data, 65 were duplicate scans, and 3 participants had no baseline diagnostic information. After quality control of WMHs, 22 cases were re-processed with the appropriate modifications, as per the re-processing step.

### BaMoS Comparison Subset

The characteristics of the 60 control and AD individuals chosen for the semi-automated comparison section of the study can be seen in online resource Table 1. There were no differences in age, race, WMH volume or gender distribution between controls and AD patients. Controls were less likely to be APOE ε4 carriers than AD participants and had significantly higher cognitive scores than AD participants.

**Table 1:** Subject demographics and basic imaging information for the ADNI cohort. Demographics are shown for controls, Early Mild Cognitive Impairment (EMCI), Late Mild Cognitive Impairment (LMCI), Subjective/Significant Memory Concern (SMC) and Alzheimer’s disease (AD). Values are mean (SD) unless stated in the table, White matter hyperintensity (WMH) is reported as median, (interquartile range). Abbreviations: Mini-mental state examination (MMSE), Clinical Dementia Rating Global score (CDRGlobal), Trails A and Trails B and Alzheimer’s disease Assessment scale cognitive subscale (ADAS-Cog)

		Controls	SMC	EMCI	LMCI	AD	Group difference (*p* value)
N		180	107	320	171	151	
Age at baseline, years		73.4 (6.2)	72.3 (5.5)	71.0 (7.5)	72.4 (7.6)	74.9 (8.0)	<0.001
Male (%)		46	43	54	56	56	0.08
Percentage APOE ε4 carriers		33	36	47	60	71	<0.001
Years of education		16.5 (2.5)	16.8 (2.5)	16.0 (2.6)	16.5 (2.5)	15.7 (2.8)	<0.001
Race(%)	Asian	1.11	0.00	1.25	0.58	3.31	0.2
	Native Hawaiian or Pacific	0.00	0.00	0.31	0.58	0.00	
	Black or African American	9.44	2.80	3.44	3.51	3.97	
	American Indian or Alaskan	0.00	0.00	0.31	0.00	0.00	
	White	87.78	94.39	91.56	94.74	91.39	
	More than one race	1.11	2.80	2.19	0.58	1.32	
	Race Unknown	0.56	0.00	0.94	0.00	0.00	
Follow up time		3.3 (1.5)	2.1 (0.9)	3.5 (1.8)	2.9 (1.6)	1.2 (0.7)	<0.001
Number of visits		5.3 (1.5)	4.1 (1.1)	5.9 (2.2)	5.5 (2.0)	3.6 (1.1)	<0.001
Baseline MMSE		29.0 (1.3)	29.0 (1.3)	28.3 (1.6)	27.6 (1.8)	23.1 (2.1)	<0.001
Baseline CDRGlobal		0 (0)	0 (0)	0.5 (0.03)	0.5 (0.03)	0.8 (0.3)	<0.001
Baseline ADAS-Cog		9.0 (4.4)	8.9 (4.3)	12.7 (5.5)	18.8 (7.2)	31.1 (8.5)	<0.001
Baseline Trails A		33.3 (10.4)	34.3 (13.0)	36.9 (14.8)	42.3(19.0)	60.8 (33.4)	<0.001
Baseline Trails B		81.8 (43.4)	86.5 (41.0)	99.0 (50)	121.6 (70.2)	195.5 (86.2)	<0.001
Baseline WMH (ml)		3.4 (4.8)	3.4 (4.4)	3.8 (6.1)	3.7 (8.1)	5.8 (9.0)	<0.001

### Neuropsychological Assessment Subset

929 participants were included in the section of the study assessing BaMoS WMH volume’s correlation with neuropsychology. Participants had a baseline scan and on average 3 to 5 cognitive assessments; the AD group had the shortest follow up time and fewer visits than the other groups (see Table 1). All groups overlapped in age, though the EMCI group were slightly younger than the other groups, and the AD group marginally older. There was no difference in sex distribution, and participants were not racially diverse, the majority were white, with the second largest group being black African Americans. As expected, prevalence of the APOE ε4 allele was greater in the EMCI, LMCI and AD groups than the SMC and control group. Baseline cognitive scores were similar between controls and SMC, and were poorer in EMCI, LMCI and AD groups. ADs had the largest volume of WMH. notably median WMH values were similar between the BaMoS comparison subset and larger set (although these comparisons were not formally tested).

### Semi-Automated Comparisons

There was a high level of agreement between semi-automated segmentations, indicated by the volumes, correlation coefficient, inter-rater reliability and overlap measures, see Table 2. The median segmentation volumes for all raters varied from the lowest of 5.62 ml (rater 4) to highest of 6.07 ml (rater 2), no significant difference in WMH volumes was detected between each segmentation compared to rater 1. A very high intraclass coefficient of 0.97 was achieved between the semi-automated segmentations, indicating good inter-rater reliability.

**Table 2:** Table comparing semi-automated segmentations between raters. Values are reported as median (inter-quartile range). Section A shows the median volumes, upper and lower quartiles of WMH volume from each rater, with (p value) showing statistical difference in each volume compared to rater 1. Inter-rater reliability (Intra-class coefficient) is shown between all raters with 95% confidence intervals. Section B of the table shows each raters performance compared to rater 1, correlation of WMH volumes using intra class correlation coefficient (ICC) with 95% confidence intervals, Dice scores of overlap, outline error false positive (OEFP) which, for a given shared WMH lesion, denotes voxels included in the segmentation which are not in the reference; outline error false negative (OEFN) which denotes, for a given shared WMH lesion, voxels which are included in the reference and not the segmentation; detection error false positive (DEFP) which denotes voxels included in the segmentation and not the reference (false positive lesions), and detection error false negative (DEFN) denoting lesions included in the reference and not the segmentation (missed lesions). Section C compares each rater to a consensus of the three remaining raters, using the metrics from section B. Statistical tests are shown for differences between each spatial metric for each rater. There were 10 controls and 10 AD patients from each of the three scanner types (Siemens, Philips and General Electric scanners)

A.	Rater 1	Rater 2	Rater 3	Rater 4	Test between raters
WMH Volume (ml)	5.70	6.07	5.96	5.62	
	(3.12–12.60)	(3.37–14.19)	(3.16–12.11)	(3.14–12.33)	
		(0.63)	(0.93)	(0.91)	
Inter-rater reliability	0.974 (0.96–0.98)				
B.	Semi- Automated Comparison to Rater 1				
ICC		0.956 (0.92–0.98)	0.998 (0.99–0.99)	0.992 (0.99–0.99)	
Dice Score		0.88 (0.84–0.92)	0.94 (0.91–0.97)	0.89 (0.87–0.93)	<0.001
OEFP		122.5 (45–407.5)	53.5 (8–147)	81 (36.5–255.5)	0.01
OEFN		49.5 (13.5–145.5)	38 (4.5–129)	61 (16.5–205.5)	0.07
DEFP		56 (31.5–89.5)	21.5 (5.5–50)	30.5 (14–52)	<0.001
DEFN		17 (6–77)	22.5 (7.5–51.5)	25 (8.5–106)	0.3
C.	Semi-Automated Comparison to Consensus				
ICC	0.997 (0.99–0.99)	0.944 (0.87–0.97)	0.995 (0.99–0.99)	0.992 (0.99–0.99)	
Dice Score	0.93 (0.9–0.95)	0.90 (0.86–0.94)	0.93 (0.89–0.95)	0.91 (0.88–0.94)	0.01
OEFP	42.5 (10–126.5)	106 (35–312)	44.5 (17.5–88.5)	76.5 (22–173.5)	<0.001
OEFN	68 (30–233)	42.5 (14.5–114)	72 (28.5–226)	65.5 (21–199)	0.07
DEFP	16 (6–73.5)	52 (30–80.5)	25.5 (10.5–80)	27 (14–46)	0.002
DEFN	23.5 (12–44.5)	13.5 (3.5–78.5)	26 (18.5–56.5)	22.5 (8–78)	0.3

Dice scores for segmentations were high compared to rater 1, ranging from a median of 0.88 (rater 1 compared to rater 2) to 0.94 (rater 1 compared to rater 3); with significantly greater overlap between raters 1 and 3, compared to 2 and 4. Dice scores of each rater compared to consensus estimates of the remaining 3 raters also showed excellent overlap, with median Dice scores of over 0.9. Further investigation of the overlap measures showed that compared to rater 1 and consensus estimates, raters 2 and 4 had a higher median OEFP and DEFP, indicating slight over segmentation compared to the reference segmentation. For rater 2 this was an OEFP of 122.5 voxels on average compared with rater 1 with analogous statistics for rater 3 of 53.5 and for rater 4 of 81 voxels. Compared with rater 1 the percentage of OEFP/FP was 64.9%, for rater 3 this was 66.2%, and for rater 4 this was 71.4%. Comparable statistics for OEFN/FN were 59.2% for rater 2 compared with rater 1, 44.9 for rater 3 compared with rater 1, and 63.1% for rater 4 compared with rater 1. For rater 2, an average DEFP of 56 voxels was calculated compared with rater 1 with analogous statistics of 21.5 voxels for rater 3 and 30.5 voxels for rater 4.

The intra-rater reliability was also high, with an intraclass coefficient of 0.98 comparing rater 1’s first and second segmentation see Table 3. A median dice score of 0.91 also showed excellent spatial overlap. In contrast to raters 2 and 4, the second segmentation of rater 1 showed a tendency to under-segment (indicated by a high OEFN), this was also reflected in the slightly lower median volume in the second segmentation compared to the first, although there was no overall significant difference in volumes between the first and second set of segmentations (*p* = 0.4).

**Table 3:** Table comparing semi-automated segmentations between rater 1’s first and second segmentation. Values are reported as median (inter-quartile range), unless stated. Section A shows the WMH volume from the first and second segmentation rounds, and (p value) showing statistical differences between these WMH volumes. Intra-rater reliability (intra class correlation coefficient) with 95% confidence intervals is reported. Section B of the table shows Dice scores of overlap, outline error false positive (OEFP) which, for a given shared WMH lesion, denotes voxels included in the segmentation which are not in the reference; outline error false negative (OEFN) which denotes, for a given shared WMH lesion, voxels which are included in the reference and not the segmentation; detection error false positive (DEFP) which denotes voxels included in the segmentation and not the reference (false positive lesions), and detection error false negative (DEFN) denoting lesions included in the reference and not the segmentation (missed lesions). There were 10 controls and 10 AD patients from each of the three scanner types (Siemens, Philips and General Electric scanners)

A.	Rater 1	Rater 1
	First segmentation	Second segmentation
Volume	5.70	5.31
	(3.12–12.60)	(2.73–11.00)
		(0.4)
Intra-rater reliability	0.976 (0.92–0.99)	
B.	Comparison to first segmentation	
Dice Score		0.91
		(0.86–0.94)
OEFP		34.5
		(12.5–100.5)
OEFN		149
		(69–373)
DEFP		7.5
		(4–24)
DEFN		24
		(9–55)

### BaMoS Comparisons

There was an excellent agreement between the automated and semi-automated volumes; with a strong correlation of volumes between BaMoS and each rater, and BaMoS and the consensus of all 4 raters, ranging from 0.88 (rater 2) to 0.96 (raters 1, 3, and consensus), see Table 4. There was no statistically significant difference in estimated volumes between the raters, consensus and BaMoS.

**Table 4:** Table comparing semi-automated segmentations from each rater, and consensus of the 4 raters, to BaMoS automated values. Values are reported as median (inter-quartile range), unless stated. Volumes from each rater, the consensus, and BaMoS are reported, with (p value) showing difference compared to BaMoS. Correlation coefficients are given for each method compared to BaMoS using intra class correlation coefficient (ICC) with 95% confidence intervals. Spatial metrics of the following are given for to compare BaMoS with each rater/consensus as the reference; Dice scores of overlap, outline error false positive (OEFP) which, for a given shared WMH lesion, denotes voxels included in the segmentation which are not in the reference; outline error false negative (OEFN) which denotes, for a given shared WMH lesion, voxels which are included in the reference and not the segmentation; detection error false positive (DEFP) which denotes voxels included in the segmentation and not the reference (false positive lesions), and detection error false negative (DEFN) denoting lesions included in the reference and not the segmentation (missed lesions). Statistical tests are shown for differences between each spatial metric for each rater. There were 10 controls and 10 AD patients from each of the three scanner types (Siemens, Philips and General Electric scanners)

	BaMoS	Rater 1	Rater 2	Rater 3	Rater 4	Semi-automated Consensus	Test (BaMoS vs raters)
Volume	5.56	5.70	6.07	5.96	5.62	5.61	
	3.88–11.18)	(3.12–12.60)	(3.37–14.19)	(3.16–12.11)	(3.14–12.33)	(2.94–11.94)	
		(0.94)	(0.58)	(0.87)	(0.97)	(0.83)	
Comparison to BaMoS							
ICC		0.958	0.875	0.958	0.944	0.959	
		(0.93–0.97)	(0.78–0.93)	(0.93–0.97)	(0.91–0.97)	(0.93–0.98)	
Dice Score		0.73	0.74	0.73	0.72	0.74	>0.9
		(0.63–0.81)	(0.66–0.81)	(0.64–0.8)	(0.66–0.8)	(0.66–0.82)	
OEFP		261.5	219.5	263	245.5	250	0.5
		(144.5–490)	(136.5–420)	(145–502)	(152.5–481)	(150–498)	
OEFN		234.5	306.5	255	260.5	226	0.3
		(114–544.5)	(147–738.5)	(124–527.5)	(121.5–640)	(120–543.5)	
DEFP		197	169	203.5	196	210	0.2
		(144.5–255.5)	(114–220.5)	(151–255)	(147.5–273)	(150–264)	
DEFN		48.5	53	47	45	26	0.8
		(11.5–147.5)	(33–120)	(13–131)	(18–108.5)	(8–73.5)	

BaMoS’ segmentations overlapped well with each rater and the consensus segmentations, with median dice scores between 0.72 and 0.74 for all the raters. There was no difference in BaMoS’ performance between the raters in any spatial comparison metric. There was also no clear pattern in outline error between BaMoS and the semi-automated segmentations; the proportion of OEFP to OE was 52.0%, suggesting that BaMoS tended to both under- and over-segment true positive lesions in roughly equal measure. As a proportion of FP, OEFP was on average 56.0% and OEFN/FN was on average 82.5%. However, DEFN (64 voxels on average compared with consensus; DEFN/FN 15.4%) was much lower than DEFP (215 voxels on average compared with consensus; 42.4% DEFP/FP). This indicated that whilst BaMoS tended not to have any issues missing WMH segmentations, the algorithm more often classified hyperintense voxels that would not be classified as lesion by a human operator as WMH than it missed lesions (DEFP/DE = 80.6%). A confusion matrix demonstrating overall results for the consensus segmentations against BaMoS is shown in Table 5.

**Table 5:** Confusion matrix showing overall differences between BaMoS and the semi-automated consensus segmentations in the 60 semi-automatically segmented individuals.

		BaMoS	
		No Lesion	Lesion
Semi-automated consensus	No Lesion	NA	35.0 (OE:22.1 / DE:12.9)
	Lesion	27.6 (OE:23.8 / DE:3.8)	114.0

Figures represent sum over 60 subjects in mls. *NA* not applicable, *OE* outline error, *DE* detection error

T1-space BaMoS segmentations had a median volume of 4.38 ml (IQR 3.10–8.32), whilst FLAIR space BaMoS had a slightly higher median volume of 5.56 ml (3.88–11.18). There was no significant difference between volumes in T1 space and T2 FLAIR space (t = 1.56, *p* = 0.12). BaMoS segmentations performed in T1 space compared well to those run in T2 FLAIR space, with a correlation coefficient of 0.87 (95%CI 0.67–0.94).

GE scanners were associated with greater WMH volumes than Philips and Siemens for both BaMoS and the semi-automated consensus, see online resource Table 2. Analyses on the log-transformed volumes showed this GE-related difference was significant. Both Siemens and GE scanners were associated with a significantly higher Dice score than Philips’ scanners. OEFP and OEFN were significantly lower for Siemens than Philips’, and significantly higher than Philips for GE scanners. There were no differences between scanner performance for DEFN, and DEFP.

### Bland Altmann

**Fig. 2:**
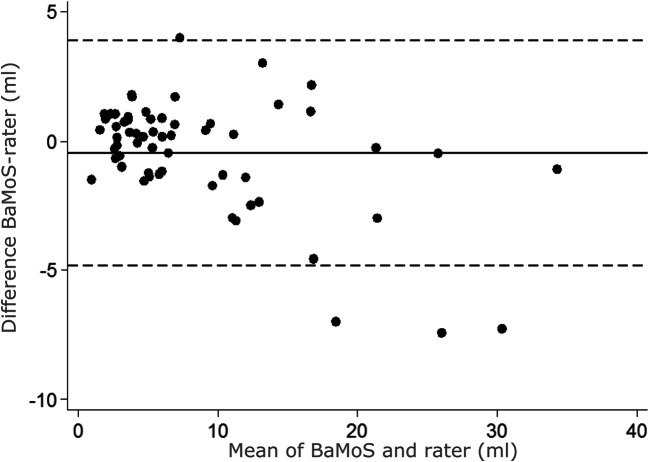
Bland Altmann of BaMoS generated WMH volumes compared to consensus of 4 raters WMH volumes. The difference between the two volumes is plotted on the y axis and the mean of the two volumes is plotted on the x axis. The mean difference between the two volumes is represented by the black line, and the 95% limits of agreement are the dotted line (mean difference ± 1.96*standard deviation of the mean difference)

The Bland Altmann plot (Fig. 2) shows a good distribution of points overall above and below the mean difference line, with the mean difference close to zero. BaMoS tends to slightly overestimate volume at small and medium loads indicated by points above the line at lower volumes (<10 ml), whereas at larger volumes BaMoS tends to underestimate WMH volume compared to rater 1 (>15 ml).

### BaMoS Comparisons by Location

**Fig. 3:**
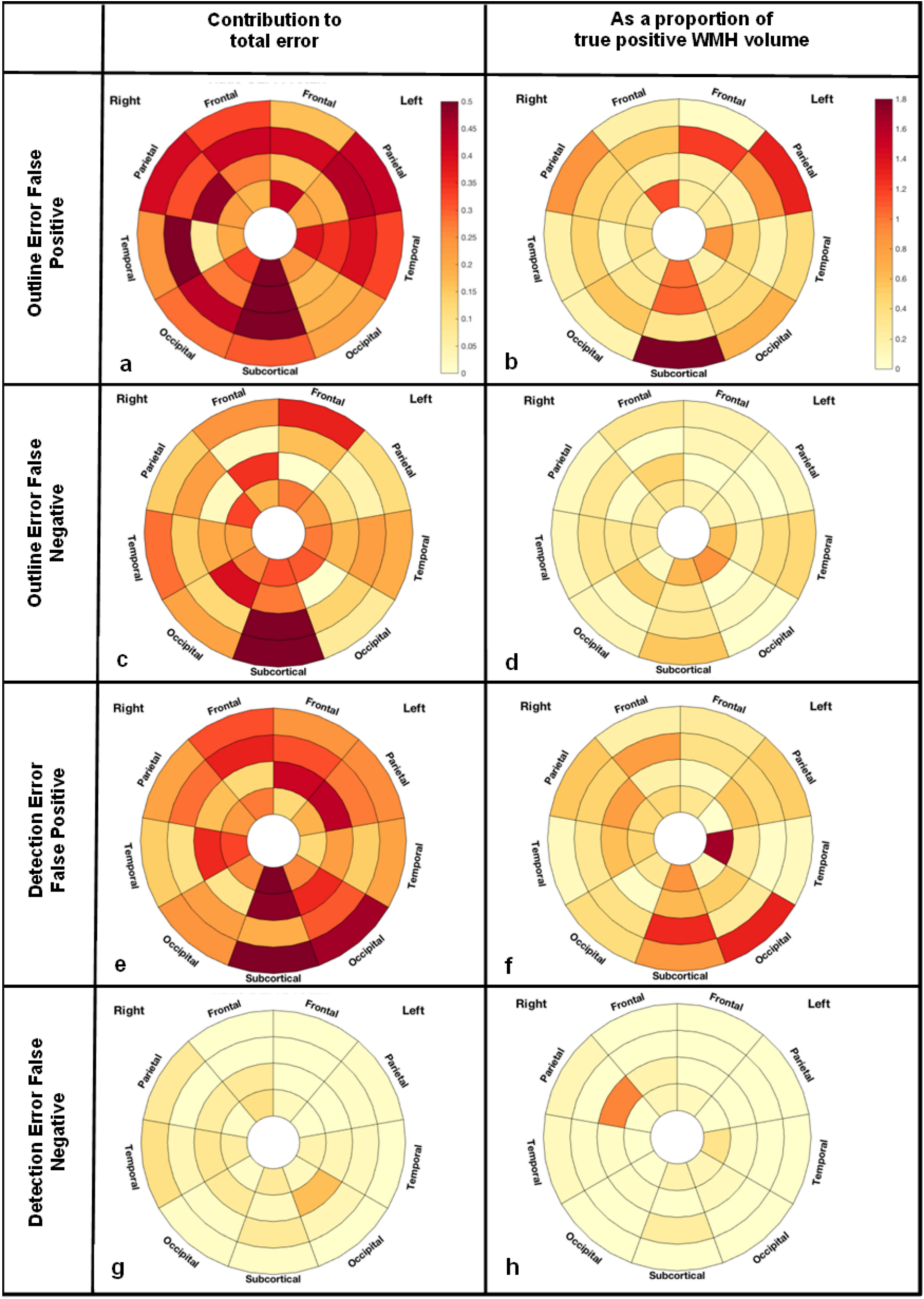
Bullseye plots showing ratios of spatial metrics as a proportion of total error (a, c, e and g) and as a proportion of true positive white matter hyperintensity (WMH) volume (b, d, f and g). Each concentric ring of the bullseye represents a cortical WM layer from each lobe, with the innermost ring representing the inner cortical layer (closest to the midline ventricles), and the outer ring representing the cortical layer nearest the grey matter. A and b report outline error false positive (OEFP) denoting voxels included in the segmentation (BaMoS) which are not in the reference (consensus). C and d represent outline error false negative (OEFN), voxels which are included in the reference and not the segmentation. Bullseyes e and f show detection error false positive (DEFP) denoting voxels included in the segmentation and not the reference (false positive lesions). g and h show detection error false negative (DEFN) denoting lesions included in the reference and not the segmentation (missed lesions)

**Fig. 4:**
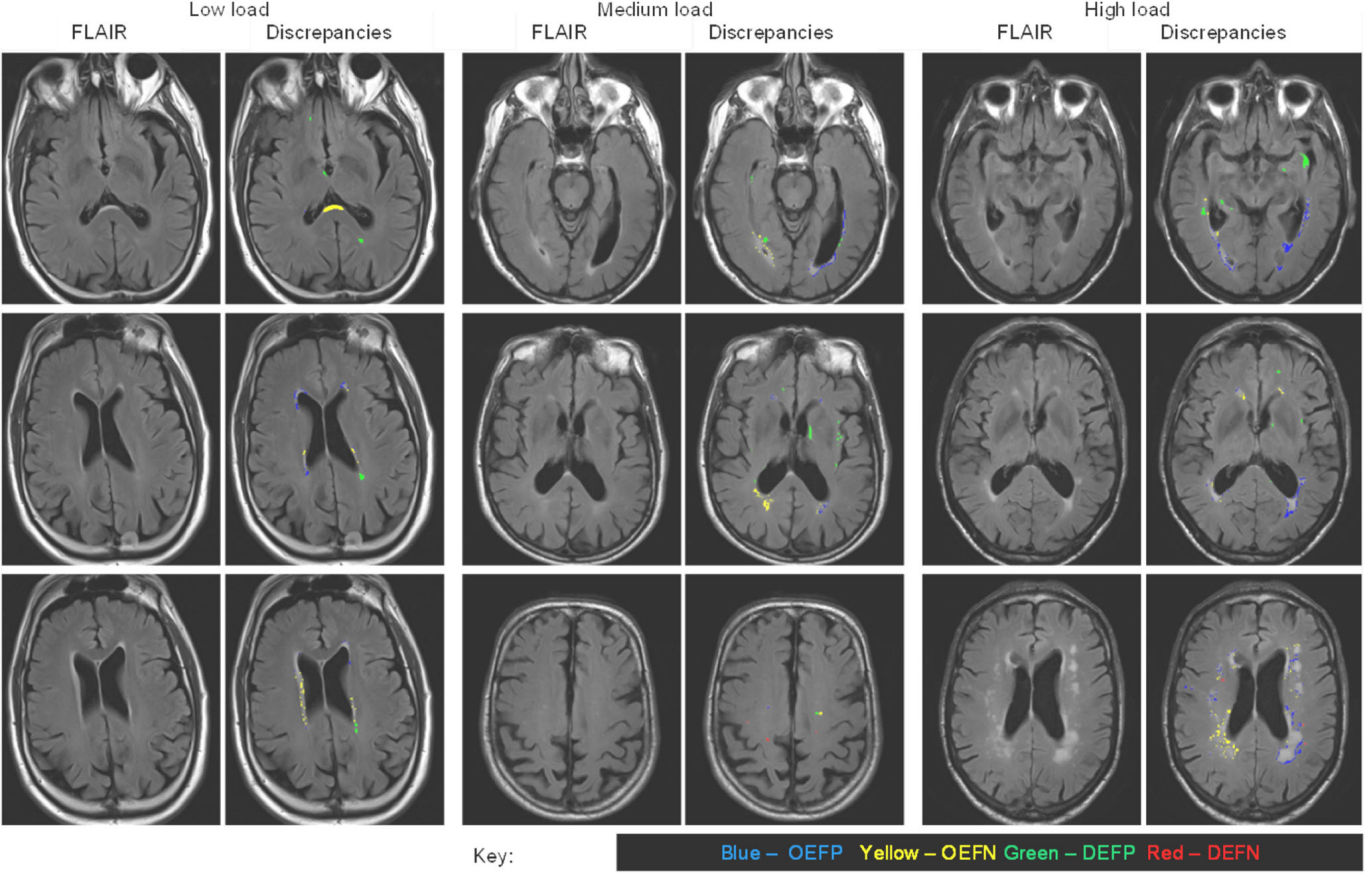
Images showing differences in spatial metrics between BaMoS automated segmentation and consensus of all 4 raters, in subjects with low, medium and high WMH loads. FLAIR images are shown in the left column, with difference maps overlaid in the right column. Blue voxels signify outline error false positive (OEFP) which, for a given shared WMH lesion, denotes voxels included in BaMoS which are not in the consensus. Yellow represents outline error false negative (OEFN) which denotes, for a given shared WMH lesion, voxels which are included in the consensus and not in BaMoS. Green represents detection error false positive (DEFP) which denotes voxels included in BaMoS and not the reference (false positive lesions). Red represents detection error false negative (DEFN) denoting voxels included as lesion in the consensus and not BaMoS

OEFP (over-segmentation of true positive lesions) is a major contributor of the total volume of error between BaMoS and consensus per individual, as indicated in Fig. 3 by bullseye a, compared to bullseyes c, e and g. This widespread association may be mainly driven by OEFP as a large proportion of total error at small volumes, because when OEFP volume is considered as a proportion of true lesion volume (bullseye b), OEFP has a reduced predominance, affecting a few regions, particularly the subcortical, parietal, and frontal regions. Observation of the difference maps (Fig. 4) shows that OEFP (in blue), is prevalent at all lesion loads, and to a larger degree in the left hemisphere (right-hand side of images and bullseyes). The periventricular caps are affected by OEFP in the low load example, perhaps explaining the bullseye association in the frontal lobes.

OEFN, denoting under-segmentation of true positive lesions by BaMoS, contributes to overall error in a few key areas pointed out by the bullseye c, namely the frontal and subcortical regions. When considering the volume of OEFN in consideration of TP volume it does not appear to be influential; suggesting that OEFN may be contributing most to error at larger lesion loads. OEFN is indicated by yellow in the difference maps and is present at all lesion volumes, particularly in the right hemisphere, which is also clear in bullseye c (left on image and on bullseye).

DEFP, denoting bright areas mistaken for lesions, contribute to error across the brain (bullseye e); however, when considered as a proportion of true lesion volume, FP appears as an issue at small volumes in the subcortical, occipital and temporal regions (bullseye f). DEFP is denoted by green in the difference maps (Fig. 4), and it is noticeable that it tends to be picked up in the subcortical regions at all loads.

DEFN, exemplifying lesions missed by BaMoS, are uncommon compared to the other error types, and hardly contribute to total error (bullseye 3 g). On the difference maps they are denoted in red. A missed lesion is apparent in right parietal lobe in Fig. 4 (left on image from medium load case and bullseye), this is indicated on the bullseye on DEFN/TP, showing this region is vulnerable over numerous participants.

### Associations of BaMoS Derived WMH to Baseline, and Change in Neuropsychology

After fitting the initial models, the residuals were calculated and plotted (using the qnorm function in Stata) to check for model fit and outliers in cognitive scores. Due to ceiling effects, residuals were skewed in all tests apart from ADAS-Cog, this affected all groups apart from AD. To counteract this skew, models were rerun using bootstrap with 2000 iterations, for controls, early MCI, late MCI and SMC groups, for MMSE, CDRGlobal, Trails A and B. Twenty-three outliers were identified from residual plots and the cognitive scores underlying the outliers were investigated for each participant. Five outliers were judged to be genuine errors in data collection or entry, with the remaining 18 outliers assessed likely to be caused by sudden deteriorations or fluctuations in cognitive abilities (see online resource Table 3). The covariance structure was changed to independent where necessary (as participant baseline score was not sufficiently correlated with participant decline in that score) in controls CDRGlobal, LMCI CDRGlobal, SMC MMSE, SMC CDRGlobal, SMC Trails A and SMC Trails B.

#### Controls

Significant deterioration in MMSE, CDRGlobal, and Trails B were detected in controls over the course of the study (see Table 6). No overall change over time in ADAS-Cog and Trails A was observed. Controls with greater WMH values performed worse on Trails A at baseline. A doubling of baseline WMH volume also predicted a greater decline in MMSE, of 0.07 points per year; this represents a 70% increase in MMSE decline compared to the average change of −0.1 points per year. A doubling of WMH volume predicted a borderline significant worsening of 0.12 ADAS-Cog points per year, compared to no overall average change.

**Table 6:** Results of the models of neuropsychological change predicted by white matter hyperintensity (log_2_WMH) volume. Values are shown as estimate (p value) [95% confidence intervals]. Models were run separately in each group; controls, early Mild Cognitive Impairment (EMCI), late mild cognitive impairment (LMCI), Subjective/Significant Memory Concern (SMC) and Alzheimer’s disease (AD). Baseline scores and change in each neuropsychology test predicted by the model are reported; Mini-mental state examination (MMSE), Clinical Dementia Rating Global score (CDRGlobal), Trails A and Trails B and Alzheimer’s disease Assessment scale- cognitive subscale (ADAS-Cog). Estimates are shown for a change in neuropsychology (baseline or change in) for a doubling of baseline WMH compared to the average baseline volume. Models are adjusted for age, sex, years of education, APOE genotype (binary covariate indicating presence of an ε4 allele). Models were bootstrapped for all groups apart from AD.

		Controls	EMCI	LMCI	SMC	AD
Baseline	MMSE	28.96	28.12	28.30	28.62	21.96
		(<0.001)	(0.00)	(<0.01)	(<0.001)	(<0.01)
		[28.65, 29.28]	[27.47, 28.76]	[27.17, 29.43]	[27.83, 29.41]	[20.53, 23.39]
	CDRGlobal	0.02	0.42	0.53	0.02	0.82
		(0.27)	(<0.001)	(<0.01)	(0.40)	(<0.001)
		[−0.02, 0.06]	[0.38, 0.47]	[0.48, 0.57]	[−0.02, 0.06]	[0.65, 0.99]
	ADAS-Cog	9.00	11.97	11.97	11.27	27.98
		(<0.001)	(<0.001)	(<0.001)	(<0.001)	(<0.01)
		[7.27, 10.74]	[9.79, 14.16]	[7.13, 16.80]	[8.21, 14.32]	[22.45, 33.51]
	Trails A	35.88	44.29	49.09	41.60	68.00
		(<0.001)	(<0.01)	(<0.001)	(<0.001)	(<0.001)
		[31.16, 40.59]	[38.14, 50.44]	[36.56, 61.63]	[30.33, 52.86]	[46.47, 89.53]
	Trails B	102.33	124.61	128.56	95.52	208.44
		(<0.001)	(<0.01)	(<0.001)	(<0.001)	(<0.01)
		[74.16, 130.50]	[102.76, 146.46]	[73.56, 183.56]	[69.22,121.82]	[155.98, 260.90]
Change in	MMSE	−0.10	−0.23	−1.12	−0.15	−2.15
		(0.01)	(<0.001)	(<0.01)	(0.01)	(<0.001)
		[−0.17, −0.03]	[−0.33, −0.14]	[−1.38, −0.86]	[−0.26, −0.03]	[−2.69, −1.61]
	CDRGlobal	0.02	−0.01	0.09	0.05	0.24
		(<0.001)	(0.25)	(<0.01)	(<0.001)	(<0.001)
		[0.01, 0.03]	[−0.01, 0.00]	[0.06, 0.12]	[0.03, 0.07]	[0.17, 0.30]
	ADAS-Cog	0.06	0.61	2.36	0.03	5.44
		(0.49)	(<0.001)	(<0.001)	(0.85)	(<0.001)
		[−0.11, 0.22]	[0.38, 0.85]	[1.79, 2.93]	[−0.30, 0.37]	[4.25, 6.63]
	Trails A	0.07	0.31	4.16	0.22	10.46
		(0.75)	(0.35)	(<0.001)	(0.74)	(<0.001)
		[−0.36, 0.49]	[−0.33, 0.95]	[2.26, 6.07]	[−1.09, 1.53]	[5.79, 15.13]
	Trails B	2.31	2.76	11.13	1.37	21.95
		(0.02)	(<0.01)	(<0.001)	(0.33)	(<0.001)
		[0.37, 4.25]	[0.94, 4.59]	[6.44, 15.83]	[−1.39, 4.14]	[10.63, 33.28]
Effect of WMH on Baseline	MMSE	−0.08	−0.02	−0.20	0.15	0.02
		(0.21)	(0.73)	(0.05)	(0.05)	(0.87)
		[−0.19, 0.04]	[−0.13, 0.09]	[−0.40, 0.01]	[0.00, 0.30]	[−0.28, 0.33]
	CDRGlobal	0.00	0.00	−0.00	0.00	0.02
		(0.45)	(0.68)	(0.64)	(0.75)	(0.37)
		[−0.00, 0.01]	[−0.01, 0.01]	[−0.02, 0.01]	[−0.01, 0.01]	[−0.02, 0.05]
	ADAS-Cog	0.03	0.18	1.06	0.58	0.10
		(0.89)	(0.44)	(0.01)	(0.04)	(0.86)
		[−0.39, 0.45]	[−0.27, 0.63]	[0.27, 1.84]	[0.02, 1.14]	[−1.06, 1.26]
	Trails A	1.37	0.63	1.26	1.03	0.57
		(0.02)	(0.34)	(0.31)	(0.19)	(0.81)
		[0.18, 2.57]	[−0.65, 1.92]	[−1.16, 3.68]	[−0.51, 2.57]	[−4.18, 5.32]
	Trails B	3.61	3.99	4.67	1.15	5.21
		(0.17)	(0.06)	(0.37)	(0.65)	(0.42)
		[−1.51, 8.72]	[−0.19, 8.18]	[−5.65, 14.99]	[−3.76, 6.07]	[−7.38, 17.79]
Effect of WMH on change in	MMSE	−0.07	−0.07	−0.08	−0.11	0.06
		(0.04)	(0.01)	(0.34)	(0.03)	(0.77)
		[−0.13, −0.00]	[−0.13, −0.02]	[−0.24, 0.08]	[−0.21, −0.01]	[−0.32, 0.44]
	CDRGlobal	0.00	0.01	0.03	−0.00	0.04
		(0.37)	(0.08)	(0.01)	(0.68)	(0.08)
		[−0.01, 0.01]	[−0.00, 0.01]	[0.01, 0.05]	[−0.02, 0.01]	[−0.01, 0.10]
	ADAS-Cog	0.12	0.18	0.22	0.01	−0.21
		(0.05)	(0.03)	(0.26)	(0.47)	(0.62)
		[−0.00, 0.24]	[0.02, 0.34]	[−0.16, 0.59]	[−0.16, 0.36]	[−1.05, 0.63]
	Trails A	0.19	0.12	0.29	0.61	−1.63
		(0.30)	(0.56)	(0.64)	(0.11)	(0.34)
		[−0.17, 0.55]	[−0.29, 0.53]	[−0.93, 1.52]	[−0.17, 1.37]	[−4.99, 1.72]
	Trails B	1.55	0.94	0.07	0.12	−1.11
		(0.06)	(0.07)	(0.96)	(0.92)	(0.80)
		[−0.09, 3.18]	[−0.09, 1.97]	[−2.68, 2.82]	[−2.25, 2.54]	[−9.91,7.69]

#### SMC

SMC participants did not show any change over time in Trails A, Trails B and ADAS-Cog, but did decline in MMSE and CDRGlobal. Increased WMH volume was borderline associated with higher MMSE score and poorer baseline ADAS-Cog score. A 0.11 points per year increase in MMSE decline was predicted in those with double the average baseline WMH volume, a 73% increase in MMSE decline compared to the average annual decline of 0.15 points per year.

#### EMCI

EMCI participants declined in performance in MMSE, ADAS-Cog and Trails B over the course of the study. No differences over time were seen in CDRGlobal and Trails A. There were no significant correlations between baseline WMH volume and baseline neuropsychology. A doubling of baseline WMH volume predicted a 0.07 point greater annualised decline in MMSE, a 30% increase in MMSE decline compared with the average MMSE decline of 0.23 MMSE points per year. A worsening of 0.18 on ADAS-Cog was observed for a doubling of WMH volume, compared to an average change of 0.61, indicating a 30% increase in decline.

#### LMCI

LMCI participants experienced decline over time in all the neuropsychological tests investigated. Greater baseline WMH volume was borderline associated with lower baseline MMSE score. A doubling of WMH volume predicted a 0.03 increase in CDRGlobal change per year, a 33% increase in CDRGlobal deterioration, compared to the average change of 0.09 CDRGlobal points per year.

#### AD

AD participants declined over time in all neuropsychology tests. Baseline WMH volume was found to be neither associated with baseline neuropsychology, nor change in neuropsychology.

### Results before Bootstrapping and Outlier Removal

Results before bootstrapping and outlier removal are seen in online resource Table 4. Results are very similar both with and without bootstrapping and before outlier removal. Outliers may be due to errors in data collection or entry (see online resource Table 3). A few minor changes were present after outlier removal and bootstrapping; with bootstrapping/outlier removal the relationship between WMH volume and change in Trails B changes to trend level from p < =0.05 for controls and EMCI. With bootstrapping/outlier removal the relationship between WMHs and baseline MMSE in LMCI and SMC changed to p < =0.05 from trend level.

## Discussion

### Findings from this Study

In this study, we assessed the performance of BaMoS, an automated approach to WMH estimation. We have found BaMoS’ WMH estimates to agree well with gold standard semi-automated segmentations, and that BaMoS’ WMH values have the ability to predict cognitive change over time in control, EMCI, LMCI and SMC individuals. The success of BaMoS’ ability to estimate volumes over several scanner types, which match well to human ratings and which are also able to predict neuropsychological test outcomes, demonstrate the algorithm’s potential to be applied to large scale data sets, inform disease processes underlying dementia, and to potentially contribute to clinical practise with further research.

### Context of Findings

WMH volumes produced by BaMoS were able to predict cognitive change over time in controls, EMCI, LMCI and SMC participants. Our results are consistent with previous studies, which have found higher baseline WMH in controls is related to lower baseline cognition (Mosley et al. 2005) in controls and greater cognitive decline (De Groot et al. 2002), as well as in a model including controls, MCI and AD subjects from ADNI phase 1 (Carmichael et al. 2010). Others have found that lesion progression is related more to progression of cognitive decline (Silbert et al. 2008; Van Dijk et al. 2008), and that WMH in combination with atrophy relates to poorer cognition (Swardfager et al. 2018; van der Flier et al. 2005). In this study WMH was also a predictor of cognitive decline in SMC individuals, as was found by Benedictus (Benedictus et al. 2015). BaMoS-generated volumes had strong predictive power for cognitive change, a doubling of WMH was associated with a > 70% increase in MMSE decline in controls and SMC. That such large effects sizes for cognitive change were predicted by automated volumes is promising; further useful insights may be gained by using BaMoS segmentations in future studies. Of note, we did not find that WMH was associated with baseline cognition nor changes in cognition in AD subjects which may appear discrepant compared with results from ADNI phase 1 (Carmichael et al. 2010). This may be due to the manner in which models are fitted, with our study fitting separate models for each diagnostic group.

BaMoS’ correlation coefficient of 0.96 and median Dice score of 0.74 indicates a good agreement compared to the semi-automated consensus segmentation; a Dice score exceeding 0.7 is considered the benchmark of a good segmentation (Caligiuri et al. 2015). This ranks BaMoS well compared to other WMH segmentation tools in the literature, although comparing Dice scores across studies is problematic due to differences between studies in scanner types, image acquisition and study populations (Caligiuri et al. 2015). Other WMH segmentation tools which have been tested in healthy control and AD populations have achieved similar average Dice scores to BaMoS; Seghier 0.64; de Boer 0.72; Yoo 0.76; Griffatini 0.76; Wang 0.78; and Yang 0.81 (de Boer et al. 2009; Griffanti et al. 2016; Seghier et al. 2008; Wang et al. 2012; Yang et al. 2010; Yoo et al. 2014). However, few studies have used data from multiple sites. Samaille et al. used information from several sites as in our study (Dice score of 0.72), and Dyrby et al. from 10 sites (Dice of 0.56) (Dyrby et al. 2008; Samaille et al. 2012). The most comparable study to ours is from Dadar et al., who also investigated ADNI2/Go participants over a range of scanner types and achieved a Dice score of 0.72, similar to our study (Dadar et al. 2017). In 2017, The Medical Image Computing and Computer Assisted Intervention Society, held a conference challenge in which segmentation algorithms were compared to manual WMH estimates, results of which ranged from Dice scores 0.23 to 0.80, with a mean score of 0.64. In this challenge a previous version of BaMoS achieved a Dice score of 0.68. Notably BaMoS is disadvantaged by the Dice metric, as larger WMH volumes lead to higher Dice scores, and BaMoS in this study has estimated a lower volume than other studies on similar participant groups; the median value in the comparison subset was just over 5 ml (5.3 ml), whilst other studies generally report values under 5 ml as at the lower end of WMH burden, with medium loads between 5 and 20 ml (Dadar et al. 2017; Griffanti et al. 2016). Taking into consideration the lower volumes estimated and multisite nature of this study, BaMoS performs excellently. Whilst BaMoS achieves a comparable WMH performance to that in the literature, the focus of this study is to demonstrate the similarity of BaMoS to human ratings of WMH and feasibility to large multisite studies and not to compare it to existing techniques, for which it has already been extensively validated (Sudre et al. 2015).

To better understand any proposed WMH segmentation method, it is important to have multiple complementary metrics to assess its performance compared to the gold standard. The Dice score alone is unable to tell us about over- or under-segmentation, nor any information about the location of errors, which is important to consider as some regions are more difficult to segment than others. We employed a variety of metrics to assess the performance of BaMoS compared to the gold standard, including spatial overlap metrics, difference maps and bullseye plots (Sudre et al. 2018). We found that whilst errors are common across the brain, they are found more in some regions due to biases in both the semi-automated protocol and BaMoS. BaMoS was most consistently vulnerable to errors in subcortical regions, both over-, and under-segmenting in this area, and more prone to false positives lesions here too. Such issues were also seen in the temporal and occipital lobes. Other problematic regions were the parietal lobes and frontal lobes, likely due to the presence of diffuse dirty white matter in the parietal lobes and difficulties segmenting periventricular caps. BaMoS also appeared to over-segment at lower volumes compared to the gold standard and under-segment at higher volumes. However, the bias towards under-segmentation at higher volumes compared to consensus may rather be an issue caused by the semi-automated segmentation protocol; thresholds for semi-automated segmentation were based on median brain intensity, in individuals with higher WMH volume the median brain intensity would be higher, therefore causing greater inclusion of borderline hyperintense voxels at higher volumes. A further systematic cause of difference between protocols may be due to bias correction; FLAIR images were bias corrected by BaMoS preceding WMH segmentation but were not viewed as bias corrected by raters during semi-automated segmentation. The bias correction difference is apparent on the left vs right hemisphere, with more included in the consensus on the left, (visible on left side of images from the highest load- Fig. 4). It is necessary to understand how and why differences with respect to human segmentation arise in order for methods to be improved.

WMH segmentation is challenging for both humans and computers. To tackle this we generated a superior gold standard, using segmentations from four raters who each segmented 60 participants. Using information from multiple raters reduced the risk of the algorithm being penalised as a result of human error. Such a rigorous generation of a gold standard is uncommon in the literature, algorithms are usually compared to segmentations from one or two raters of 20–30 participants (Anbeek et al. 2004; Beare et al. 2009; Wang et al. 2012; Yoo et al. 2014). Some authors have generated superior gold standards; Griffatini used 3 raters, and Admiraal Beehoul and Dyrby segmented a larger proportion of the dataset (100% in the case of Dyrby) (Admiraal-Behloul et al. 2005; Dyrby et al. 2008; Griffanti et al. 2016). Raters in this study agreed well in their WMH estimates, achieving mean Dice scores of 0.90–0.93 and mean correlation coefficients of 0.994–0.997 when comparing each rater to consensus estimates. Although efforts were taken to make the semi-automated segmentation as objective as possible, each rater naturally developed subtle tendencies to include or exclude WMH in their segmentation. There were similarities between raters which reflected who taught whom the segmentation protocol. Raters 1 and 3 were highly similar, whilst raters 2 and 4 were most similar; rater 1 trained 3 (and 2), and rater 2 trained rater 4. Differences arose because raters 2 and 4 were more likely to include WMH, than raters 1 and 3. These individual differences demonstrate the inherent difficulty of the WMH segmentation, the need for a well generated gold standard, and the need for frequent retraining of manual segmentors, even when using a structured protocol incorporating semi-automated thresholding.

Direct comparison between semi-automated and automated methods is hindered by differences in T1-weighted and T2 FLAIR image resolution; semi-automated segmentation occurs in FLAIR space, whilst the algorithm segments in T1-weighted volumetric space. FLAIR space is preferable for human rater segmentation because WMH are more clearly visible on FLAIR than on T1, and resampling issues arise when the thicker slice FLAIR is registered to the T1. However, the most accurate automated segmentations can be obtained in T1 space. The segmentations used in the neuropsychology assessment were generated by BaMoS in T1 space, whilst FLAIR space segmentations were used for method comparison. BaMoS’ volumes in FLAIR space correlated well with T1 space segmentations, so it is valid to assume that they are functionally equivalent, and that our assessment of FLAIR space segmentations is relevant to those generated in T1 space. Differences between methods were also found to be scanner dependent; individuals scanned with GE Scanners had higher WMH volumes and greater differences in outline error (OEFP, OEFN) than Philips and Siemens. It is difficult to pinpoint the reason for the observed difference; GE scanners may have a hyperintense bias present which leads to greater inclusion of WMH, or they may be more sensitive to a particular lesion type which is poorly detected by Philips and Siemens. Notably, the semi-automated protocol recognised a general hyperintensity in GE images in the posterior white matter, and raised the thresholds for segmentation for this scanner type compared to Philips and Siemens. Interestingly, individuals scanned with a GE scanner had higher WMH volumes detected by both BaMoS and the semi-automated segmentation, indicating both methods classified the increased hyperintensity as lesion, withstanding the bias correction unique to BaMoS, and the higher thresholds implemented for GE scanners from the semi-automated protocol. FLAIR has not been as widely used as T1-weighted MRI sequences, especially for quantitative analysis. Increased research using FLAIR, and volumetric FLAIR, in the coming decade, will likely progress our knowledge of differences between scanner types, understanding of the pathology underlying hyperintense signal, and improve comparability between semi-automated and automated methods.

### Strengths of this Work

The strengths of this paper lay notably in the existence of multiple rater WMH estimates that enabled the comparison of a total of 300 segmentations from 60 participants. We developed the protocol for semi-automated segmentation in collaboration with UMC Utrecht. No harmonisation of protocols exist for WMH classification, as has been achieved with hippocampal segmentations (Boccardi et al. 2015). More research is required to validate WMH protocols across institutions. Further, we investigated the performance of BaMoS using a number of comparison metrics that allowed a greater understanding of the origins of discrepancies between methods, and where differences were most likely to occur. We studied participants scanned at multiple sites, allowing the algorithm to be assessed using images from multiple scanners. We did not adjust for multiple comparisons in tests of WMH and cognition, as our tests were hypothesis driven and answering different questions. We adjusted for multiple covariates in analyses with cognitive outcomes, such as race, years of education, age, sex and APOE ε4 status, as per Carmichael et al (Carmichael et al. 2010), however, we did not adjust for cardiovascular risk. Whether head size is related to change in cognition is yet unresolved and we did not adjust for this covariate in our analyses. Some studies have suggested larger head size is protective against cognitive decline (Guo et al. 2013; Perneczky et al. 2010), another provides opposing evidence for head size effect on atrophy rate (Fiford et al. 2017). How head size associates with cognition is a complex question related to theories of cognitive reserve, which deserves thorough investigation. Since the relationship of WMH to cognition is well-established (Prins and Scheltens 2015), and the purpose of this study was to assess how WMH volumes from a novel automated technique relate to cognitive change, we did not investigate total intracranial volume (head size) effects. We noted that the subcortical area was most prone to discrepancies which may be explained by a lower signal-to-noise ratio in this region.

### Limitations of this Work

The semi-automated segmentation set consisted only of controls and AD patients; any difference in the performance of the algorithm that may exist in other diagnostic groups could therefore not be tested. We appreciate this investigation included only older controls and groups considered to be potentially prodromal/preclinical AD or clinical AD subjects. Therefore, our study is not an assessment of the ability of BaMoS to segment WMH in other diseases (such as neuroinflammation) or in paediatric cases. Additionally, although not a goal of our work, it is important to appreciate that the ADNI population is not sufficiently ethnically diverse to understand the confounding effect of ethnicity on the WMH-cognitive relationship. We used Dice scores as one statistic to describe spatial overlap of segmentations. The Dice score is useful to evaluate the delineation of anatomical structures where the range in size and shape is relatively limited and is a widely used metric for validation of segmentation frameworks. However, for pathological lesions such as WMH, the Dice score is imperfect due to its dependence on shape and size of targeted elements. The cost of a single-voxel error decreases when the overall size of the object to segment increases leading to higher Dice scores in datasets with higher lesion loads. To alleviate the Dice limitations, distance metrics (such as Hausdorff or Average distance) are often used in conjunction to provide a different perspective on the performance. These metrics are however not really applicable in the case of very low out-of-plane resolution. Instead, we chose statistics which were appropriate for the thick-slice FLAIR imaging data we used in this study and tried to describe precisely the distribution of disagreements.

We note that whilst BaMoS did predict cognitive decline in controls, EMCI, LMCI, SMC, it did not do so in AD subjects. Whether this reflects that WMH in AD is inherently different and truly less related to cognitive decline, or whether cognitive decline in AD is driven by other factors, or whether BaMoS was less accurate in this large AD sample is unclear. We only considered WMH where there was hyperintensity on FLAIR and some evidence of hypointensity on T1. Other pathologies of the white matter were not included in this study. The semi-automated segmentation used thresholds that were determined relative to median brain intensity. The reported values in this publication may not be suitable for other studies where the acquisition protocol is very different or the disease or age groups are very different to those included here. In contrast, for the automated segmentation, since the thresholds are based on measures of outlierness with respect to healthy tissues, the default values can be used over a wide range of acquisition settings. Diffusion imaging can be used to identify specific tracts and artefacts associated with those tracts. However, only a proportion of individuals (GE scanners only) had diffusion imaging in ADNI2/Go so the assumptions made about hyperintense areas being associated with tract-based artefacts, such as those commonly found in the corticospinal tracts, may be incorrect. We did not assess the accuracy of segmentations according to lesion type (such as diffuse lesions vs. punctate lesions where partial volume characteristics may differ). This may be a further area for assessment and algorithmic development.

### Conclusions

In conclusion, we have assessed the performance of BaMoS and found it matches very well to human generated WMH segmentation methods, and to be predictive of change in neuropsychology scores in controls, SMC, EMCI and LMCI. Our method was meticulously compared to ‘gold standards’ and found to perform well over multiple sites and scanners. We can therefore confidently apply BaMoS to large-scale multi-site studies, and, with more research, this algorithm may be of potential clinical use.

## Information Sharing Statement

Data used in this study were obtained from the Alzheimer’s Disease Neuroimaging Initiative (ADNI) database (RRID:SCR_003007,http://www.loni.usc.edu/). ADNI data is freely available to research groups.

## Electronic Supplementary Material

ESM 1 (PDF 772 kb)
