# Supplementary material for: Automated White Matter Hyperintensity Segmentation Using Bayesian Model Selection: Assessment and Correlations with Cognitive Change
Source: Neuroinformatics. 2020 Feb 15;18(3):429–49. doi: 10.1007/s12021-019-09439-6 (PMC7338814; doi:10.1007/s12021-019-09439-6)
Supplement: Supplementary file 1 — (PDF 772 kb) [file 12021_2019_9439_MOESM1_ESM.pdf]

Supplementary Material - Automated White Matter Hyperintensity Segmentation using Bayesian Model Selection: assessment and correlations with cognitive change

Cassidy M. Fiford[a,*], Carole H. Sudre[b,a,c,*], Hugh Pemberton[a], Phoebe Walsh[a], Emily Manning[a], Ian B. Malone[a], Jennifer Nicholas[d], Willem H Bouvy[e], Owen T. Carmichael[f], Geert Jan Biessels[e], M. Jorge Cardoso[b,a,c,*], Josephine Barnes[a,*], for the Alzheimer's Disease Neuroimaging Initiative[1]

[a] *Dementia Research Centre, Department of Neurodegenerative Disease, UCL Queen Square Institute of Neurology, London, UK.*
[b] *School of Biomedical Engineering and Imaging Sciences, King's College London, London, UK*
[c] *Centre for Medical Image Computing, Department of Medical Physics and Biomedical Engineering, University College London, London, UK*
[d] *London School of Hygiene and Tropical Medicine, London, UK*
[e] *Department of Neurology and Neurosurgery, Brain Center Rudolf Magnus, University Medical Center Utrecht, the Netherlands*
[f] *Pennington Biomedical Research Center, Baton Rouge, LA, USA*

List of softwares used

The following software and packages have been used for the completion of this work:

Registration tools NiftyReg: open source code available at `https://github.com/KCL-BMEIS/niftyreg` used for the coregistration of T1 and FLAIR images as well as part of the parcellation pipeline

Segmentation tools NiftySeg: open source code available at `https://github.com/KCL-BMEIS/niftyseg` used for the skull-stripping, the parcellation, the subject-specific priors (BaMoS) the brain mask (semi-automated segmentation)

---

*Authors contributed equally

[1] Data used in preparation of this article were obtained from the Alzheimer?s Disease Neuroimaging Initiative (ADNI) database (adni.loni.usc.edu). As such, the investigators within the ADNI contributed to the design and implementation of ADNI and/or provided data but did not participate in analysis or writing of this report. A complete listing of ADNI investigators can be found at: `http://adni.loni.usc.edu/wp-content/uploads/how_to_apply/ADNI_Acknowledgement_List.pdf`

Visualisation tools NiftyMidas: open source code available as part of the NiftK package `https://github.com/NifTK/NifTK` used for the segmentation assessment and the semi-automated segmentation.

BaMoS WMH segmentations were run with the default initialisation parameters: degree 3 for the bias field correction, initial level of outlierness (0.1), outlierness definition (Mahalanobis distance > 3)

Comparison Package Creation of bullseyes plots - Evaluation metrics between segmentations - Open source code available at `https://github.com/csudre/EvaluationCharacterisation`

Statistical Analysis Stata SE v13 (Stata Corp.)

Definition of similarity measures

In the following, $\sharp$ represents the cardinality of a set, while $\overline{S}$ refers to the complement of a set S.

TP True positives. Number of voxels classified both in Seg and Ref $TP = \sharp(Seg \bigcap Ref)$

FP False positives. Number of voxels segmented in the Seg but not in the Ref. $FP = \sharp(Seg \bigcap \overline{Ref})$

FN False negatives. Number of voxels segmented in the Ref but not in Seg. FN=$\sharp(\overline{Seg} \bigcap Ref)$

DSC Dice Score Coefficient $DSC = \dfrac{2TP}{2TP + FN + FP}$

TPc Connected true positives. Number of lesion elements (connected components) that have a non void overlap between Ref and Seg.

FPc Connected false positives. Number of lesion elements that are only present in Seg (but not in Ref).

FNc Connected false negatives. Number of lesion elements that are only present in Ref (but not in Seg).

OE The outline error (OE) is defined as the number of voxels that are not common to both segmentation but that belong to a TPc element. (i.e the segmentations share for this component some voxels but there are disagreements for others)

OEFP Outline error false positives. Number of element in the outline error that are false positives. $OEFP = \sum_{L \in TPc} \sharp(L \bigcap \overline{Ref})$

OEFN Outline error false negatives. Number of element in the outline error that are false negatives.$OEFP = \sum_{L \in TPc} \sharp(L \bigcap \overline{Seg})$

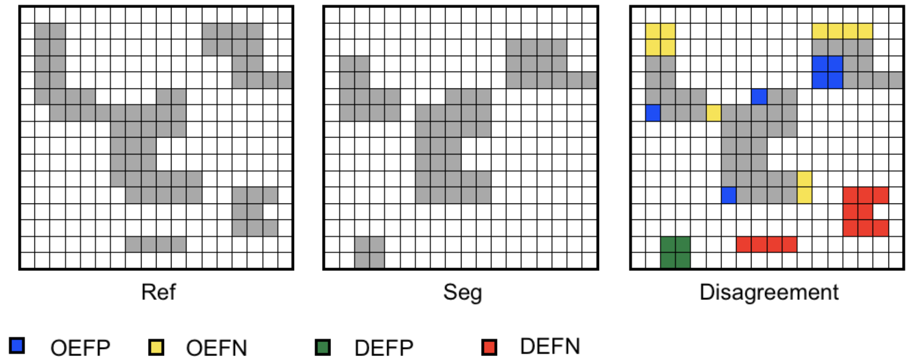

Supplementary Figure 1: Illustration of error subtypes; OEFP=Outline error false positive, OEFN=Outline error false negative; DEFP= detection error false positive; DEFN= Detection error false negative; Ref= reference segmentation; Seg= test segmentation. For a given shared WMH lesion OEFP denotes voxels included in the test segmentation which are not in the reference. OEFN denotes, for a given shared WMH lesion, voxels which are included in the reference and not the test segmentation. DEFP denotes voxels included in the test segmentation and not the reference (false positive lesions), and DEFN denotes lesions included in the reference and not the segmentation (missed lesions).

DE Detection error. Number of voxels that belong to FPc or FNc, i.e. number of elements that belong to connected elements either totally false positive or totally false negatives.

DEFP Detection error false positives. Number of voxels in the detection error that are false positives. $DEFP = \sum_{L \in FP_c} \sharp L$

DEFN Detection error false negatives. Number of voxels in the detection error that are false negatives. $DEFN = \sum_{L \in FN_c} \sharp L$

Supplementary Table 1: Subject demographics and basic imaging information for the subset which were semi-automatically segmented. Demographics are shown for controls and Alzheimer's disease (AD). Values are meaning (SD) unless reported, White matter hyperintensity (WMH) is reported as median, (interquartile range). Abbreviations: Mini-mental state examination (MMSE), Clinical Dementia Rating Global score (CDRGlobal), Trails A and Trails B and Alzheimer's disease Assessment scale - cognitive subscale (ADAS-Cog).

		Controls	AD	Group difference (p value)
	N	30	30	
	Age at baseline, years	75.0 (5.4)	75.4 (7.2)	0.8
	Male (%)	50	60	0.3
	Percentage APOE ε4^	47	73	0.03
	Years of education	16.0 (2.6)	16.0 (2.6)	>0.9
Race (%)	Black or African American	7	0	0.2
	White	93	100	
	Follow up time	3.6 (0.9)	1.5 (0.6)	<0.001
	Number of visits	5.7 (0.6)	4.0 (0.7)	<0.001
	Baseline MMSE	28.6 (1.4)	23.0 (2.0)	<0.001
	Baseline CDRGlobal	0 (0)	0.8 (0.3)	<0.001
	Baseline ADAS-Cog	10.3 (5.1)	32.5 (9.1)	<0.001
	Baseline Trails A	34.8 (12.7)	68.7 (33.3)	<0.001
	Baseline Trails B	85.4 (36.1)	212.7 (92.1)	<0.001
	Baseline WMH (ml)	3.6 (4.8)	5.3 (8.0)	0.2

Supplementary Table 2: Methods comparison differences between scanner types. Estimates of Dice score, Outline Error False Positive (OEFP), Outline Error False Negative (OEFN), Detection Error False Positive (DEFP), Detection Error False Negative (DEFN) are given for each scanner type as an estimate [95% confidence intervals]. Models were adjusted for rater. † Different from Philips ($p<0.05$) ˆDifferent from Siemens ($p<0.05$). Volumes from the consensus and BaMoS are given as median, with [first and third quartile], tests of differences between scanners were performed on log transformed (base 2) WMH values. For a given shared WMH lesion OEFP denotes voxels included in the segmentation which are not in the reference. OEFN denotes, for a given shared WMH lesion, voxels which are included in the reference and not the segmentation. DEFP denotes voxels included in the segmentation and not the reference (false positive lesions), and DEFN denotes lesions included in the reference and not the segmentation (missed lesions). Reference segmentation is the segmentation from each rater, (rater 1, rater 2, rater 3, rater 4 or consensus).

	Philips	Siemens	GE
Volume (consensus)	5.32<br>[2.67, 1.17]	5.55<br>[3.06, 9.35]	7.38†^<br>[3.00, 14.3]
Volume (BaMoS)	5.74<br>[2.72, 9.64]	5.45<br>[3.90, 9.29]	7.69†^<br>[4.36, 14.98]
Dice Score	0.66<br>[0.63,0.70]	0.76†<br>[0.73, 0.80]	0.74†<br>[0.70, 0.77]
OEFP	329.5<br>[242.2, 416.8]	186.6†<br>[99.3, 273.9]	402.7^<br>[315.4, 490.0]
OEFN	627.5<br>[434.1, 820.8]	406.4†<br>[213.0, 599.8]	831.0†^<br>[637.6, 1024.4]
DEFP	188.5<br>[158.4, 218.7]	189.0<br>[158.9, 219.2]	165.1<br>[134.9, 195.2]
DEFN	92.6<br>[57.9, 127.3]	82.3<br>[47.7, 117.0]	99.5<br>[64.9, 134.2]

Supplementary Table 3: Neuropsychology test scores at each visit for 5 subjects with outliers which were judged to be due to errors in data collection or entry. Residuals and the test score they correspond to are shown in red. All neuropsychology test scores are given for context (to show that these are likely true errors, as scores on other cognitive tests on the same day do not concur). Residual corresponds to trails A for Control and LMCI outliers (RIDs, 4060; 4491; 4114), for SMC outliers, residuals are from errors in trails B (RID 5202; 5244). Numerous residuals were identified that were deemed likely to represent true fluctuations in cognitive performance which are not shown here (n=18). LMCI= late mild cognitive impairment; SMC= subjective memory concern; RID= Respondent identifier (study subject number)

Visit	MMSE	Total 13	Trails A	Trails B	CDR	Residual
**Control Outlier 1 (RID=4060)**						
**Screening**	30				0	
**Baseline**		10	27	80		-19.09
**Month 6**	30	9	36	74	0	-11.19
**Month 12**	29	11	31	79	0	-17.08
**Month 24**	30	11	**115**	112	0	**65.05**
**Month 48**	26	13	40	135	0	-13.61
**Control Outlier 2 (RID=4491)**						
**Screening**	30				0	
**Baseline**		12	30	147		-9.16
**Month 6**	30	14	**65**	62	0	**26.13**
**Month 12**	30	6	33	57	0	-5.54
**Month 24**	30	7	33	63	0	-4.89
**Month 48**	29	14	33	110	0	-3.59
**LMCI Outlier 1 (RID=4114)**						
**Screening**	28				0.5	
**Baseline**		17	30	75		-10.82
**Month 6**	27	21	30	90	0.5	-9.88
**Month 12**	27	21	**97**	77	0.5	**57.66**
**Month 24**	27	27	22	85	0.5	-16.40
**Month 36**	27	22	33	84	0.5	-4.41
**Month 48**	25	25	29	107	0.5	-7.05
**Month 60**	24	23	34	111	0.5	-0.92
**SMC Outlier 1 (RID=5202)**						
**Screening**	30				0	
**Baseline**		10	36	74		-12.42
**Month 6**	29	10	26	63	0.5	-25.69
**Month 24**	29	11	31	**173**	0.5	**75.54**
**Month 36**	29		31	74		-27.82
**SMC Outlier 2 (RID=5244)**						
**Screening**	30				0	
**Baseline**		5	24	**300**		**161.63**
**Month 6**	30	1	43	79	0	-55.29
**Month 24**	30	2	31	50	0	-71.74

Supplementary Table 4: Results of the models of neuropsychological change predicted by white matter hyperintensity (log2WMH) volume for the models which were not bootstrapped. Values are shown as estimate (p value) [95% confidence intervals]. Models were run separately in each group; controls, early Mild Cognitive Impairment (EMCI), late mild cognitive impairment (LMCI), and Subjective Memory Concern (SMC). Baseline scores and change in each neuropsychology test predicted by the model are reported; Mini-mental state examination (MMSE), Clinical Dementia Rating Global score (CDRGlobal), Trails A and Trails B and Alzheimer's disease Assessment scale- cognitive subscale (ADAS-Cog). Estimates are shown for a change in neuropsychology (baseline or change in) for a doubling of baseline WMH compared to the average baseline volume. Models are adjusted for age, sex, years of education, APOE genotype (binary covariate indicating presence of an $\epsilon 4$ allele).

		Controls	EMCI	LMCI	SMC
Baseline	MMSE	28.95 (<0.001) [28.54, 29.37]	28.16 (<0.001) [27.63, 28.68]	28.35 (<0.001) [27.03, 29.68]	28.51 (0.00) [27.60, 29.43]
	CDRGlobal	0.02 (0.28) [-0.02, 0.06]	0.42 (<0.001) [0.38 - 0.47]	0.49 (<0.001) [0.41, 0.57]	0.02 (0.70) [-0.06, 0.09]
	ADAS-Cog	9.00 (<0.001) [7.27, 10.74]	11.97 (<0.001) [9.79, 14.16]	11.97 (<0.001) [7.13, 16.80]	11.27 (<0.001) [8.21, 14.32]
	Trails A	36.20 (<0.001) [31.77, 40.62]	43.42 (<0.001) [37.89, 48.95]	48.37 (<0.001) [35.35, 61.38]	41.32 (<0.001) [32.21, 50.42]
	Trails B	103.34 (<0.001) [86.96, 119.73]	124.61 (<0.001) [104.73, 144.50]	128.56 (0.00) [77.20, 179.92]	95.09 (<0.001) [64.76, 125.42]
Change in	MMSE	-0.09 (0.02) [-0.16, -0.01]	-0.23 (<0.001) [-0.31, -0.15]	-1.12 (<0.001) [-1.36, -0.88]	-0.14 (0.03) [-0.27, -0.01]
	CDRGlobal	0.02 (<0.001) [0.01, 0.03]	-0.01 (0.24) [-0.01, 0.00]	0.09 (<0.001) [0.06, 0.12]	0.05 (<0.001) [0.03, 0.07]
	ADAS-Cog	0.06 (0.49) [-0.11, 0.22]	0.61 (<0.001) [0.38, 0.85]	2.36 (<0.001) [1.79, 2.93]	0.03 (0.85) [-0.30, 0.37]
	Trails A	0.06 (0.77) [-0.36, 0.49]	0.31 (0.19) [-0.16, 0.77]	4.08 (<0.001) [2.46, 5.70]	0.16 (0.80) [-1.11, 1.43]
	Trails B	2.00 (0.03) [0.22, 3.78]	2.76 (<0.001) [1.34, 4.18]	10.72 (<0.001) [6.95, 14.49]	0.56 (0.76) [-3.03, 4.17]
Effect of WMH on Baseline	MMSE	-0.08 (0.20) [-0.19, 0.04]	-0.02 (0.75) [-0.13, 0.09]	-0.20 (0.06) [-0.41, 0.01]	0.14 (0.11) [-0.03, - 0.32]
	CDRGlobal	0.00 (0.45) [-0.00, 0.01]	0.00 (0.63) [-0.01, 0.01]	-0.00 (0.63) [-0.02, 0.01]	0.00 (0.80) [-0.01, 0.02]
	ADAS-Cog	0.03 (0.89) [-0.39, 0.45]	0.18 (0.44) [-0.27, 0.63]	1.06 (0.01) [0.27, 1.84]	0.58 (0.04) [0.02, 1.14]
	Trails A	1.36 (0.02) [0.25, 2.46]	0.63 (0.31) [-0.58, 1.85]	1.20 (0.26) [-0.90, 3.30]	1.03 (0.24) [-0.68, 2.74]
	Trails B	3.61 (0.10) [-0.74, 7.95]	3.99 (0.06) [-0.12, 8.11]	4.67 (0.27) [-3.70, 13.05]	0.09 (0.98) [-5.88, 6.06]
Effect of WMH on change in	MMSE	-0.07 (0.01) [-0.12, -0.01]	-0.07 (0.01) [-0.13, -0.02]	0.08 (0.34) [-0.24, 0.08]	-0.11 (0.04) [-0.21, -0.00]
	CDRGlobal	0.00 (0.37) [-0.00, 0.01]	0.01 (0.08) [-0.00, 0.01]	0.03 (<0.001) [0.01, 0.05]	-0.00 (0.77) [-0.02, 0.14]
	ADAS-Cog	0.12 (0.05) [-0.00, 0.24]	0.18 (0.03) [0.02, 0.34]	0.22 (0.26) [-0.16, 0.59]	0.01 (0.47) [-0.16, 0.36]
	Trails A	0.24 (0.13) [-0.07, 0.56]	0.12 (0.45) [-0.19, 0.43]	0.33 (0.55) [-0.74, 1.40]	0.61 (0.22) [-0.39, 1.60]
	Trails B	1.55 (0.02) [0.22, 2.88]	0.94 (0.05) [-0.02, -1.90]	0.07 (0.96) [-2.47, 2.61]	1.02 (0.48) [-1.77, 3.82]
